# Monitoring Cholera Transmission in Zambia Using the Cholera Elimination Scorecard

**DOI:** 10.4269/ajtmh.26-0057

**Published:** 2026-08-27

**Authors:** Caroline C. Chisenga, Lilian Lamba, Charlie C. Luchen, Ashley Duncan, Nyuma Mbewe, Masuzyo Zyambo, Maisa Kasanga, Fraser Liswaniso, Harriet Ng’ombe, Michelo Simuyandi, Amanda K. Debes, Roma Chilengi, David A. Sack

**Affiliations:** ^1^ Centre for Infectious Disease Research in Zambia, Lusaka, Zambia;; ^2^ Zambia National Public Health Institute, Lusaka, Zambia;; ^3^ Johns Hopkins Bloomberg School of Public Health, Baltimore, Maryland, USA;; ^4^ Emergency Preparedness and Response Cluster, World Health Organization, Lusaka, Zambia;; ^5^ Centre for Epidemic Response and Innovation, Stellenbosch University, Stellenbosch, South Africa

## Abstract

We used the cholera elimination scorecard to better understand the changing cholera patterns in Zambia. The method used surveillance data from the Zambia National Public Health Institute to identify districts that had eliminated cholera or continued to be endemic between 2015 and 2024. Between 2017 and 2022 the number of cholera-endemic districts decreased from 27 to 6; however, in 2023 and 2024, a severe outbreak occurred in Lusaka and expanded to neighboring districts. Concurrently, a separate outbreak occurred in the northern areas of Zambia. We found the cholera elimination scorecard to be a practical and simple to use tool to understand changes in cholera transmission, including identifying persistently endemic districts and districts that had relapsed after having eliminated cholera. We suggest that the cholera elimination scorecard can supplement the Priority Areas for Multisectoral Interventions methodology for countries attempting to eliminate cholera.

## INTRODUCTION

The seventh pandemic of cholera came to Africa in 1970 and to Zambia in 1977^1^ and continues as an endemic threat throughout sub-Saharan Africa.^2^ From 2022, cholera spread more widely in southern Africa, resulting in large outbreaks in the area.^3^ As part of this region, Zambia shares borders with several countries with high rates of cholera, including the Democratic Republic of the Congo (DRC), Tanzania, Malawi, Mozambique, and Zimbabwe, so it is vulnerable to the cross-border spread of cholera.^4^

In 2017, the Global Task Force for Cholera Control (GTFCC) established goals of reducing cholera deaths by 90% and eliminating cholera from at least 20 countries by the year 2030.^5^ The ambitious goal of eliminating cholera in countries in which cholera has been endemic will require considerable effort, but so far there has been little progress toward this goal. Although the GTFCC goal is a global goal, disease prevention and health care are the responsibility of each nation; thus, the actions of each country will be critical to achieving this goal.

Sustained elimination of cholera will require considerably improved water, sanitation, and hygiene (WASH) infrastructure, but the oral cholera vaccine (OCV) is also an important prevention tool pending WASH improvements. Although many countries are cholera-endemic, individual districts in the country may have different levels of risk. The GTFCC recommends identifying the areas of highest risk called Priority Areas for Multisectoral Interventions (PAMIs), where increased efforts are needed, especially including WASH and OCV.^6^ By focusing limited resources on these districts or wards identified as PAMIs, the affected countries and public health specialists worldwide hope these resources will have greater effectiveness in controlling cholera. Identification of these high-risk PAMI areas uses district- or ward-level cases per month over a period of several years (generally 5 or more years) to determine the districts with the highest average annual rates and the most persistence, as well as other risk factors. Thus, the areas identified as PAMIs tend to have a higher cholera burden over time, even though there may be differences from year to year.

In 2024, The Zambia National Public Health Institute (ZNPHI) carried out an analysis to identify PAMIs in Zambia and found that 240 wards across 54 districts in each of the 10 provinces were considered PAMIs.^1^ About 5.1 million people (28% of the population) were living in these areas, and more than half of them were living in or near Lusaka. An earlier hot spot analysis also identified areas at higher risk, most of which correspond to the areas identified as PAMIs.^7^

Although the PAMI method is very useful in identifying areas where increased control efforts are needed, this method is less helpful when attempting to monitor year-to-year changes in cholera patterns. To develop a method that would show these changes, we developed the “cholera elimination scorecard,” which can be used to monitor annual changes in cholera patterns. The method was first used in Uganda and the scorecard showed the year-by-year reduction in Uganda in the number of endemic districts (from 36 to 6) between 2018 and 2024.^8^ Cholera reports from Zambia also showed a reduction in the number of cholera cases between 2018 and 2022, but starting in late 2023 Zambia experienced a major epidemic that appeared to start in Kanyama subdistrict and then spread through many wards of Lusaka.^9^ Subsequently, cholera spread through other provinces in Zambia. Because of these contrasting cholera patterns, we used the scorecard method to understand patterns of cholera transmission in Zambia between 2017 and 2024.

The purpose of this paper, thus, is to describe the use of the cholera elimination scorecard method as applied to Zambia, show how this method provides a tool with which to monitor progress toward national cholera elimination and highlight districts that relapsed. Additional investigation will be needed to determine the factors that led to the relapses so that improved interventions can be developed.

## MATERIALS AND METHODS

The reports published in the Weekly Epidemiological Record by the WHO were used to determine the cholera burden in countries bordering Zambia for the years 2015 to 2024. To gather data for the cholera elimination scorecard, we obtained district-level cholera surveillance data from the ZNPHI for each year from 2015 to 2024. Each year, this data was examined to determine the number of cholera cases that were suspected and confirmed in that district that year or during the previous 2 years. If there were no confirmed cases during this 3-year interval, cholera was deemed to have been “eliminated” from that district. If confirmed cases of cholera were reported during at least 1 of the 3 years, the district was deemed to be “endemic.” This procedure was repeated for each of the years between 2017 and 2024 to prepare maps showing the districts that had eliminated cholera and those considered to be endemic. When a district that had eliminated cholera again reported cases, this recurrence was termed a “relapse.” The scorecard method does not require the number of cases or the incidence of cholera in its calculations, only the presence or absence of cholera in the district each calendar year.

To further explore the occurrence of cases during the 2023 and 2024 outbreak and the spread of cholera in Zambia, we prepared district-level maps of Zambia to show the districts with suspected and confirmed cases occurring each month during this epidemic period when cholera rates were markedly changing.

## RESULTS

The number of cholera cases in Zambia, Mozambique, Malawi, and Zimbabwe reported to the WHO between 2015 and 2024 is shown on Figure 1. As noted in the figure, beginning in 2022 there was a remarkable increase in the number of cases in southern Africa. The increase started in Malawi in 2022 and appeared later in Mozambique. Both Malawi and Mozambique reached a peak number of cases in 2023 (32,530 in Malawi and 39,101 in Mozambique). The epidemic started later in Zambia and Zimbabwe and peaked in 2024 (23,380 in Zambia and 20,632 in Zimbabwe).

**Figure 1.**
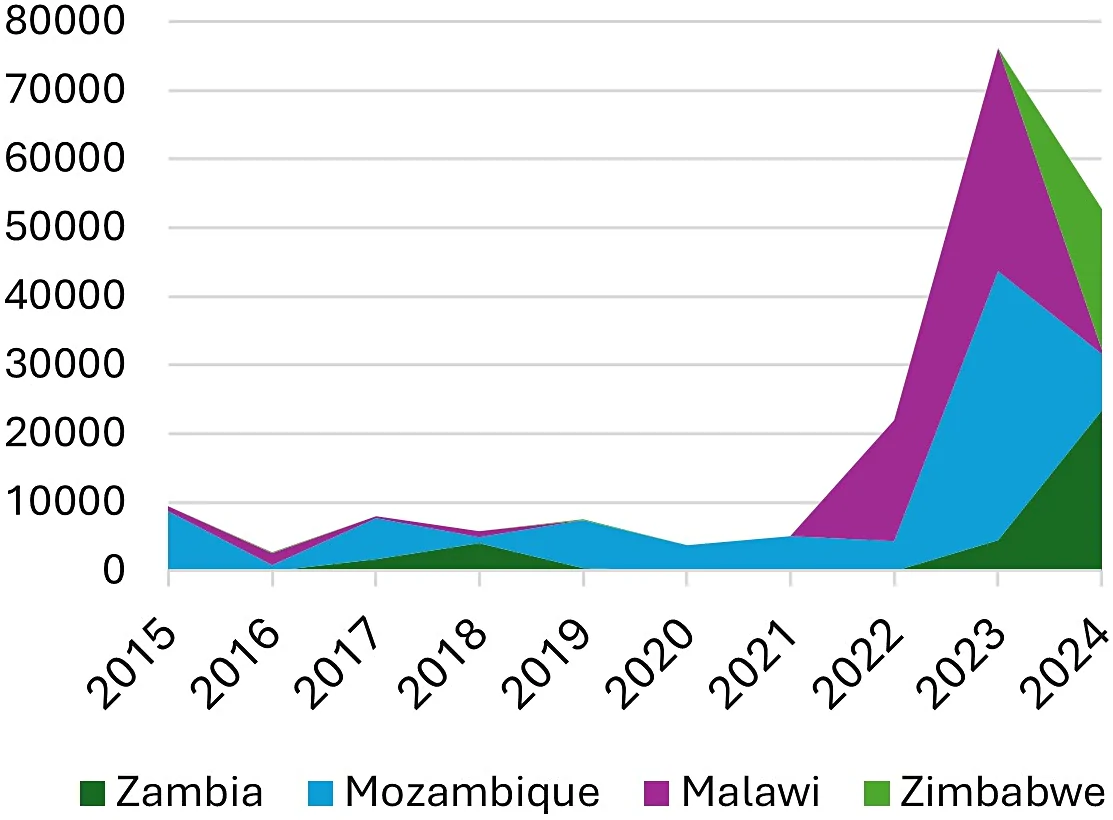
Graph depicting cholera cases in southern Africa (2015–2024) for Zambia, Mozambique, Malawi, and Zimbabwe, highlighting the increase first in Malawi, followed by Mozambique and then Zambia and Zimbabwe.

Using the scorecard method, Table 1 shows the number of districts that were deemed to be endemic, to have eliminated cholera, or to have relapsed. As shown, 18 of the 116 districts were endemic in 2017, and this number remained relatively constant between 2018 and 2020 but decreased markedly during 2021 and 2022 down to six districts. Among the districts reporting cholera, there were 20 instances when a single case was identified in a district during the year. Of the 20 instances, in eight cases, cholera was reported in the same district either the year before or the year after the single report. Of note, four of the six districts that continued to be endemic in 2022 were in Lusaka province or were close to Lusaka in the Southern province. The locations of the endemic and eliminated districts from 2018 to 2024 are shown in Figure 2.

**Table 1:** Number of districts in Zambia deemed to be endemic for cholera, having eliminated it, and relapsed between 2017 and 2024

District Status	2017	2018	2019	2020	2021	2022	2023	2024
Endemic	18	26	30	24	8	6	25	71
Eliminated	98	90	86	92	108	110	91	45
Relapsed	0	11	5	0	0	3	19	47

**Figure 2.**
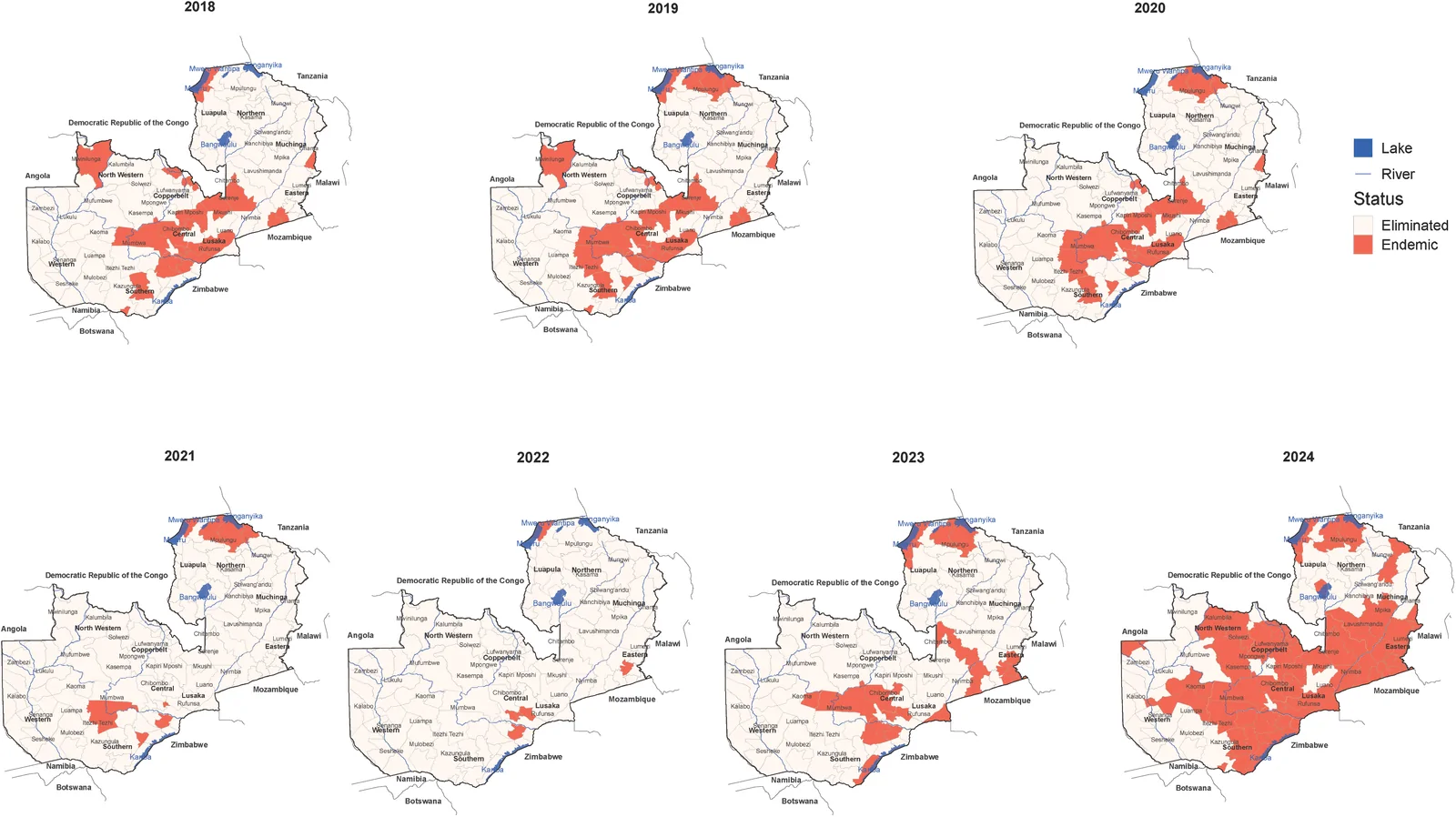
Series of Zambia maps (2018–2024) illustrating districts classified as cholera-endemic over the years.

In February 2023, cases were reported in districts near the Democratic Republic of the Congo (DRC) and Tanzania border in eastern Zambia, but these did not appear to spread to neighboring districts away from the border, and this outbreak died out by June 2023. However, in October 2023, cases began in the Lusaka Province as well as the border with Malawi, and between October 2023 and June 2024, Zambia reported 25,952 cholera cases. The Lusaka outbreak expanded rapidly southward to the Southern province and northward to the Central Province and then further north to Copperbelt Province, eventually affecting more than half (71 of 116) of the districts in Zambia. The cases in the Eastern province experienced the same time course as those in Lusaka but appeared to be separate from the Lusaka outbreak.

Figure 3 shows the districts reporting cholera each month during the epidemic period starting in September 2023 and continuing to April 2024. Although the outbreak involved most of the districts in Zambia, the highest number of cases (20,424) occurred in Lusaka province. Provinces close to Lusaka also reported large numbers of cases including Central (1,744), Copperbelt (2,160), and Southern (932) provinces. The provinces in the eastern part of the country did report cholera but with lower numbers, including Luapula (2), Northern (51), Muchinga (8), and Eastern (472). The intensity of the epidemic is illustrated in the heat map shown in Figure 4.

**Figure 3.**
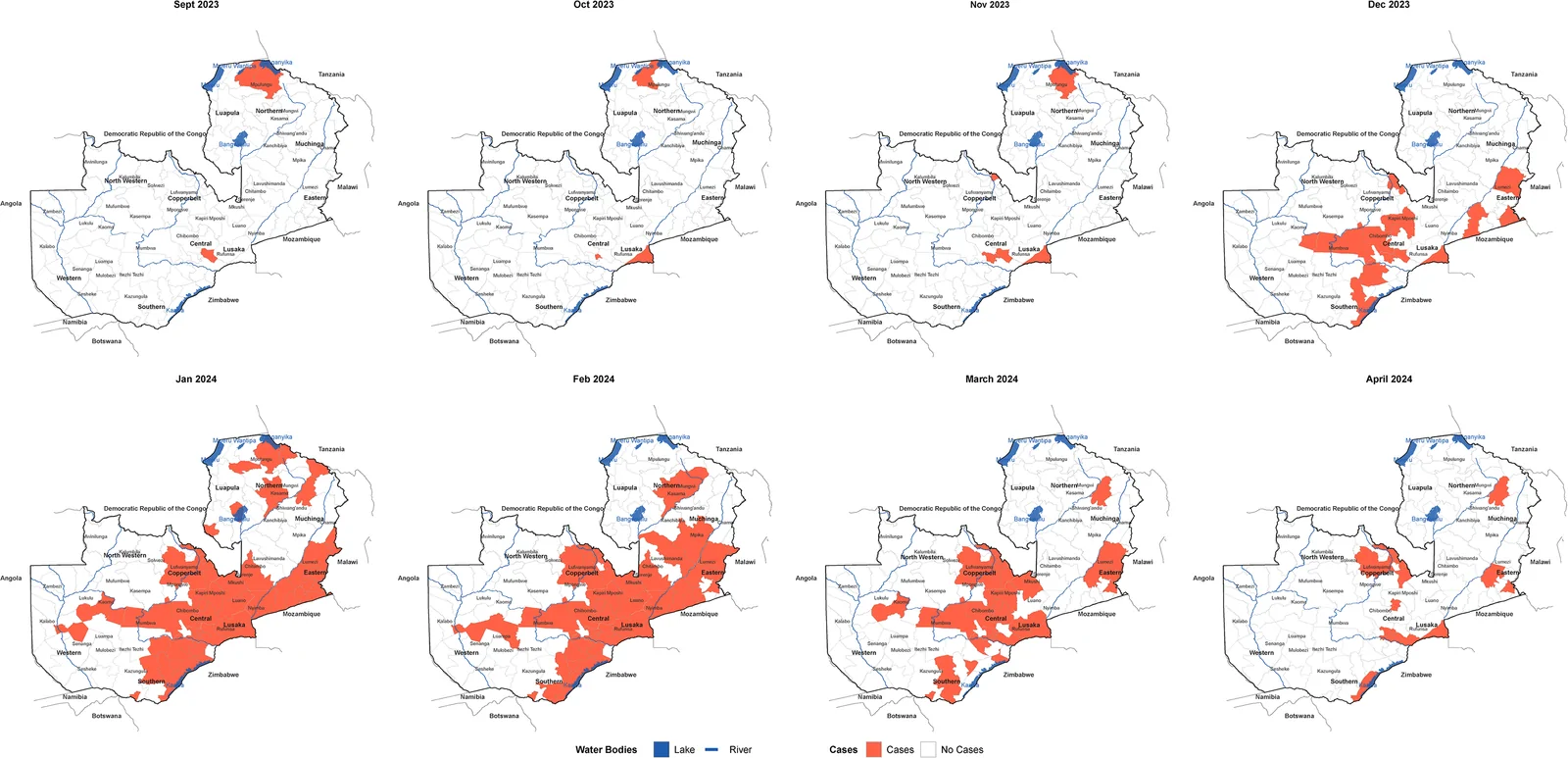
Series of Zambia maps by month (September 2023 to April 2024) showing districts reporting cholera each month, emphasizing the rapid spread of cholera between districts during this epidemic.

**Figure 4.**
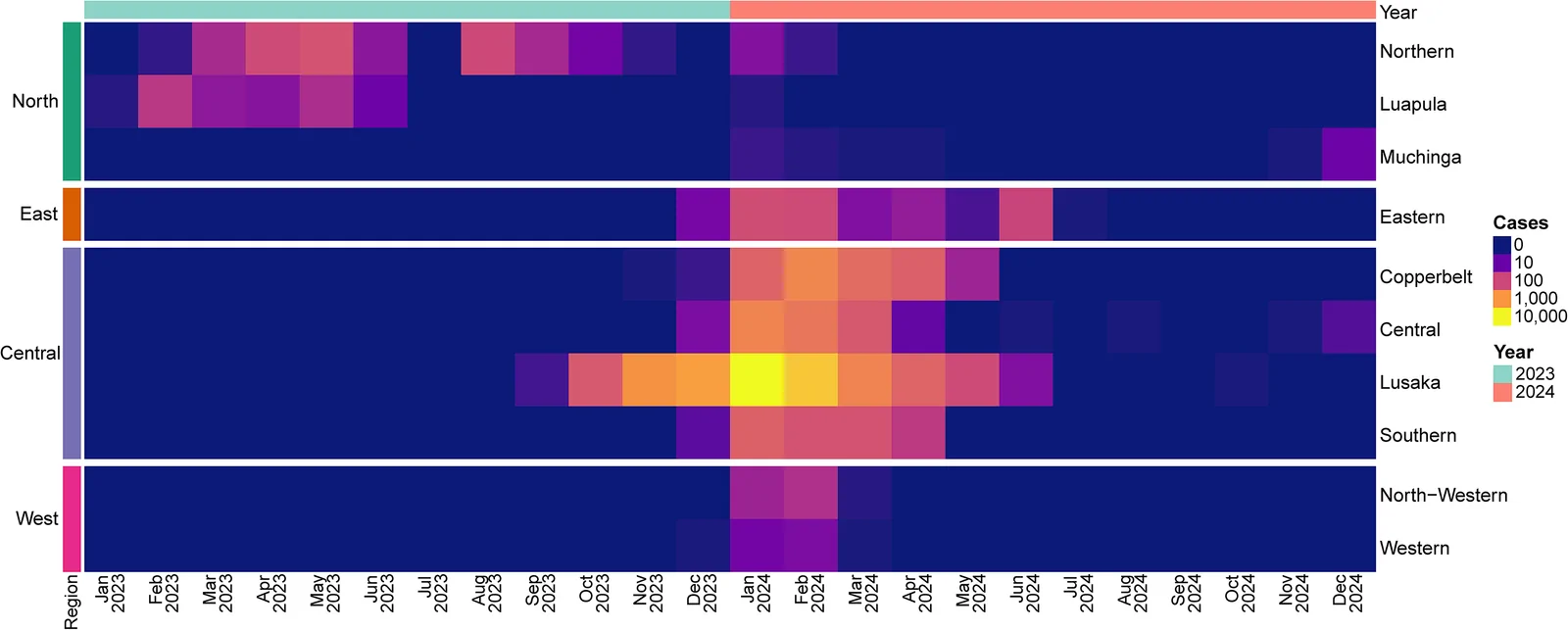
Heat map showing the number of cases each month (January 2023 to December 2024) in each of the provinces, emphasizing the high concentration of cholera cases in Lusaka and neighboring provinces.

## DISCUSSION

Zambia has been endemic for cholera for many years but is actively working toward its elimination. During most of the last 10 years, the numbers of cases in Zambia had been relatively low except for 2019 and 2020, and the scorecard maps showed progress toward elimination since the number of districts considered to be endemic decreased from 27 down to 6 in 2022. However, in 2023 and especially in 2024, many of the districts that had eliminated cholera relapsed and some of the provinces experienced large numbers of cases. The number of cases and the number of districts both show the extent of the cholera epidemic in Zambia between October 2023 and May 2024.

The scorecard maps demonstrated cholera’s persistence and consequently continued transmission in a few pockets in 2022, especially in Lusaka province, and these were the same districts from which the outbreak appeared to expand in late 2023 and then further expanded in 2024. This expansion is more clearly shown in the month-by-month maps and the heat map during 2023 and 2024, which shows the outward cross-district transmission.

The month-by-month maps show two separate outbreaks, one originating and expanding from Lusaka and another in the northeast that had more limited expansion with shorter duration. The monthly maps of these outbreaks suggested that the Lusaka outbreak may have resulted from a continued endemic focus, in or near Lusaka, whereas the northeast outbreak resulted from cross-border transmission. The endemic focus in Lusaka that expanded outward suggests that an intervention, e.g., intensive preventive OCV campaigns in the endemic Lusaka districts, may prevent future outbreaks in Lusaka and prevent the type of expansion that resulted from the large Lusaka outbreak.

When determining whether a country had eliminated cholera, according to the GTFCC definition, a country will have eliminated cholera if there were “no reports of confirmed cases with evidence of local transmission for at least three consecutive years and has a well-functioning epidemiologic and laboratory surveillance system able to detect and confirm cases.”^10^ The WHO also provides a definition for endemic cholera: “An endemic area is an area where confirmed cholera cases, resulting from local transmission, have been detected in the last 3 years. An area may be any subnational administrative unit including state, district, or smaller locality such as a ward. Any country having one or more subnational administrative units that are endemic, as defined above, is considered a cholera-endemic country.”^11^ The scorecard is thus more closely aligned with the WHO definition because it refers to elimination from subnational units over a 3-year period.

When comparing the PAMI and scorecard maps, the two approaches should not be seen as competitive, but rather complementary. The PAMI analysis for Zambia used data to identify trends in patterns of cholera cases, deaths, and vulnerability factors over an 8-year period, although the severe epidemics in 2023 and 2024 with the very large number of cases those years contributed greatly to the overall number of cases analyzed during the 8-year period. The PAMI method does not attempt to show year-to-year changes in cholera patterns but rather to identify wards that are at high risk where additional resources are needed for long term cholera prevention. The district-to-district spreading of cholera is an epidemiological feature not usually stressed in describing cholera’s epidemiology using the PAMI method. In contrast, the scorecard method does show year-to-year changes and highlights smaller areas (districts) that continue to be endemic while other districts have eliminated cholera. A reduction in the number of endemic districts suggests progress toward countrywide elimination, and the scorecard maps illustrate transmission between districts. Thus, the scorecard can show the impact of specific interventions such as preventative vaccine campaigns that would not be seen using the PAMI method.

A major difference between the two methods is the simplicity and low cost of the scorecard, which can be easily completed rapidly without the need for outside consultants or extensive training. Although it is possible that artificial intelligence might be used in the future to develop PAMI reports, the methodology for PAMI, if repeated yearly, will show minimal change from year to year and will not show impact of recent interventions.

A suspected contributing factor to the cholera epidemic in Zambia and southern Africa was the severe drought in 2023 and 2024 that triggered widespread internal migration. According to the International Organization for Migration (IOM), more than 200,000 Zambian people were displaced^12^ and traveled to areas with relatively better rainfall in search of water, food, and grazing land or to urban areas hoping for improved economic opportunities. In addition to the movement of people who may have spread the infection, the drought and migration likely also caused families to use drinking water that was less safe than their normal sources.

Clearly, the Zambian epidemic in 2023 to 2024 was part of a larger epidemic in southern Africa. This outbreak was caused by transmission of a new genetic lineage of *Vibrio cholerae*, known as T15, which had been detected earlier in Pakistan.^13^^,^^14^ It is not known whether this strain had special virulence properties or whether this lineage was especially well adapted to the severe climate events in the region. In the past, isolates related to lineage T10 and T13 have also been detected in Zambia.^15^^–^^17^ T10 is generally found in DRC and T13 in East Africa,^18^^–^^20^ and the common borders with DRC and Tanzania suggest that these lineages can also result in cross-border transmission.

When comparing Zambia with Uganda where the scorecard was used previously,^8^ Uganda appears to have eliminated endogenous cholera; that is, all the recent outbreaks have been the result of cross-border spread from a neighboring country, and there have been no outbreaks related to maintenance of cases within the country. In contrast, Zambia experiences persistent local endemic cholera pockets in addition to its vulnerability to cross-border spread from neighbors like DRC, Tanzania, Mozambique, and Malawi.^21^ When conditions favor cholera transmission, cholera can spread from these endemic pockets as well as from outbreaks due to cross-border spread. In addition to the provision of preventive measures like OCV, WASH, and rapid responses to outbreaks, enhanced surveillance in the districts of Lusaka province and the border using rapid diagnostic tests (RDTs) would allow for very early detection of cases so that the rapid response could be even more effective. Uganda has also implemented a policy of single-dose doxycycline for all confirmed cholera patients and their households with the intent of limiting onward spread.^22^

There are limitations to the cholera elimination scorecard map. For example, the scorecard does not include a measure of intensity of cholera to monitor progress toward elimination. It is possible that successful interventions could reduce the rate of cholera from year to year, and this improvement may represent progress toward elimination, but this would not be evident using the scorecard. Because rates of cholera in endemic areas often vary from year to year, such reductions in rates would have to be sustained over many years before one could be certain of progress.

A second limitation relates to the use of routine surveillance data. Cases may be undercounted because of limitations of the surveillance system itself, and cases may also be overcounted when all diarrhea cases are reported as cholera during an outbreak. Because the scorecard uses only the presence or absence of cases in a district during the year, the overcounting and undercounting present lesser problems for the scorecard method.

Another uncertainty occurs when a solitary case is reported from a district during a year. We found 20 instances in which a solitary case was reported from a district in a given year during this 10-year surveillance period. Clinically, a single case during a year in a district is very rare and calls into question whether these were true cases. However, the scorecard includes only confirmed cases, and it seems likely that other cases may have been unreported, so we have continued to treat these as true cases. In the future, we expect that a more expanded use of RDTs will improve detection of cases and when combined with confirmation should improve the reliability of the surveillance system and the scorecard.^23^

Another limitation relates to the assurance that cholera has truly been eliminated from a district because there could be viable *V. cholerae* O1 persisting in environmental water, but the current definitions for elimination only include monitoring for clinical infections. This concern may need to be reassessed as methods for environmental detection of *V. cholerae* O1 are enhanced.

## CONCLUSION

As a complement to the PAMI, we feel the cholera scorecard may be a practical tool with which to monitor changes in cholera patterns, allowing countries to monitor progress toward elimination and identify districts that relapse. When districts relapse, reasons for the relapse should be investigated in hopes of identifying ways to prevent these reoccurrences in the future. Finally, the scorecard may also identify persistent pockets of endemicity where more focused efforts may need to be targeted.
